# Healthy adult gut microbiota sustains its own vitamin B12 requirement in an *in vitro* batch fermentation model

**DOI:** 10.3389/fnut.2022.1070155

**Published:** 2022-12-01

**Authors:** Palni Kundra, Annelies Geirnaert, Benoit Pugin, Paola Morales Martinez, Christophe Lacroix, Anna Greppi

**Affiliations:** Laboratory of Food Biotechnology, Department of Health Sciences and Technology, Institute of Food, Nutrition and Health, ETH Zürich, Zurich, Switzerland

**Keywords:** cobalamin, human gut microbiota, SCFA, *in vitro* fermentation, prototrophy

## Abstract

Vitamin B12 (cobalamin) is present in the human lower gastrointestinal tract either coming from the unabsorbed dietary fraction or from *in situ* production of the gut microbiota. However, it is unclear whether the gut microbial communities need exogenous B12 for growth and metabolism, or whether B12 in low and high levels could affect gut community composition and metabolite production. Here, we investigated *in vitro* B12 production of human fecal microbiota and the effects of different levels of B12 (as cyanocobalamin) on composition and activity. Eight fecal communities from healthy human adults distributed over three enterotypes, dominated by *Firmicutes* (*n* = 5), *Bacteroides* (*n* = 1) or *Prevotella* (*n* = 2) were used to perform batch fermentations in Macfarlane medium supplemented with low B12 medium (Control, 5 ng/ml, within the tested fecal range), no B12 addition (NB12), and high B12 addition (ExtraB12, 2500 ng/ml). The microbiota community composition (qPCR, 16S rRNA metabarcoding), metabolic activity (HPLC-RI), and B12 levels (UHPLC-DAD) were measured after 24 h incubation at 37^°^C under strict anaerobic conditions. All fecal microbial communities produced B12 in the NB12 condition after 24 h, in the range from 152 ± 4 to 564 ± 25 ng/ml. None of the B12 treatments had an impact on total bacterial growth, community richness, diversity and total metabolite production, compared to the low B12 control. However, a significant increase of propionate was measured in ExtraB12 compared to NB12. Most taxonomic and metabolite changes compared to control incubations were donor-dependent, implying donor-microbiota-specific changes upon B12 treatments. Our *in vitro* data suggest that healthy human adult gut microbial communities have the capacity to produce B12 at levels fulfilling their own requirements, independently of the initial B12 content tested in the donor’s feces. Further, supplementation of exogenous dietary B12 may have limited impact on the healthy human gut microbial community composition and function.

## Introduction

The human gut microbiota forms a dense ecosystem whose composition and metabolic activity, e.g., production of Short-Chain Fatty Acids (SCFA) from undigested food components, have been extensively correlated with human health and diseases (1, 2). Thus, many efforts in microbiome research have been directed toward understanding undigested nutrient metabolism and the discovery of effective means for modulating the microbiome through the diet to improve health.

B-vitamins are essential micronutrients for humans and for the majority of microbes (3), and are associated with enzymatic activity as co-factors for numerous cellular reactions involving energy production, neurotransmitter synthesis, immune functions, and one-carbon metabolism (4). Vitamin B12 (cobalamin; referred here as B12), the precursor of the B12 coenzyme family, is a class of water-soluble molecules with a complex organometallic structure containing a corrin ring bound to a central cobalt atom, to which upper and lower ligands are attached (5). The upper axial ligand consists of a cyano-, methyl-, adenosyl-, or hydroxyl-functional group. The most stable form of B12 is cyanocobalamin, which is mostly utilized for food fortification or in dietary supplements (6). B12 is involved in vital cellular processes such as the synthesis of DNA, tRNA, glutamate and methionine (5). Furthermore, in microbes, B12 is involved in pathways leading to SCFA production, as a cofactor for methylmalonyl-CoA mutase to produce propionate, and in one carbon metabolism (Wood–Ljungdahl route) leading to the production of acetate (5, 7, 8).

Humans consume B12 either from dietary intake or from supplements, which are widely sold at high cyanocobalamin concentrations ranging from 100–5000 μg (9, 10). About 50% of B12 consumed with a normal diet is absorbed in the small intestine, though this absorption decreases with increasing dose, with only ∼1.3% absorption at a high dose of 1000 μg (11). The unabsorbed B12 fraction reaches the colon and is available to the gut microbial community, together with the locally produced B12 by prototrophic bacteria [i.e., all Fusobacteria, and specific *Bacteroidetes* and *Firmicutes* members (12)]. However, there is still no clear consensus on whether the gut microbiota depends on exogenous B12 to maintain the community composition and functionality, or whether B12 cross-feeding between prototrophic and auxotrophic bacteria is sufficient to fulfill the community needs. Finally, it remains unclear to what extent B12 is produced by gut microbial communities, as no studies have investigated B12 production by cultivating complex human gut microbiota in the absence of B12.

Here, we studied the B12 production capacity of healthy human fecal gut microbiota and the effect of deprivation or excess of B12 (as cyanocobalamin) on community growth, gut microbiota community, and metabolite production. We used fecal samples from eight human adults with different microbial and metabolic profiles and varying fecal B12 content to perform strict anaerobic batch fermentations with different B12 concentrations. Changes in the gut community composition and metabolites were compared to the control MacFarlane medium, selected to mimic the human proximal colon chyme, and containing low B12. Furthermore, we quantified the extracellular and intracellular B12 levels in our samples before and after incubation to investigate the B12 uptake of gut microbial communities.

## Materials and methods

### Fecal sample collection

Fecal samples from eight healthy adult donors [six female (D1, D3, D5, D6, D7, and D8) and two male (D2 and D4); median age of 29 years old; western style diet] who fulfilled the following inclusion criteria: good general health with no symptoms of chronic or autoimmune disease, a regular eating pattern (3 meals/day, 5 days/week), regular bowel movements (once every 3 days to 3 times per day), no use of immune suppressing drugs, blood thinners, antibiotics, or other medication affecting the gut transit and/or digestion, no intake of pre- and probiotics during the month preceding the sample donation, no regular intestinal discomfort, no operative intestinal interventions, and not being on a weight loss diet were used for the study.

Fecal sample was collected directly after defecation using a sterile container and an anaerobic atmosphere generator (Anaerogen, Thermo Fisher Diagnostics AG, Pratteln, Switzerland) and transported within 1 h into an anaerobic chamber (10% CO_2_, 5% H_2_, and 85% N_2_; Coy Laboratories, Grass Lake, MI, US). A 20% w/v fecal suspension was prepared in sterile anaerobic peptone water (0.1%, pH 7; Thermo Fisher Diagnostics AG). The suspension was homogenized by vortexing in the presence of sterile 5 mm glass beads, let stand for 5 min to allow particles to settle to the bottom. The resulting supernatant was removed with sterile pipettes and transferred to an autoclaved anaerobic Serum flask and used for batch inoculation. The ethics committee of ETH Zurich exempted this study from review because the sample collection procedure was not performed under conditions of intervention, and it was carried out in an anonymized manner.

### Batch fermentations

Brown serum flasks (20 ml, Infochroma AG, Goldau, Switzerland) containing 10 ml of sterile anaerobic Macfarlane medium adapted for batch fermentation conditions [two fold buffering capacity; pH 7.0; composition described in Bircher et al. (13)] were used. The Macfarlane medium was previously developed from the analysis of intestinal content to mimic the human proximal colon chyme of Western adults (14). This medium includes the addition of a filter-sterilized vitamin solution (15), leading to a final vitamin concentration of (ng/ml medium): folic acid, 20; cyanocobalamin, 5; pyridoxine-HCl, 100; 4-aminobenzoic acid, 50; nicotinic acid, 50; biotin, 20; thiamine, 50; riboflavin, 50; phylloquinone, 0.075; menadione, 10; pantothenate, 100 (all purchased from Sigma-Aldrich Chemie GmbH, Buchs, Switzerland). Vitamins were weighted in the dark and the vitamin solution was filtered sterilized through 0.2 μm nylon membrane filter (Infochroma AG), covered by aluminium foil and stored at 4^°^C before being supplemented to the autoclaved fermentation medium.

Each fecal microbial suspension was grown in three different media containing distinct levels of cyanocobalamin: (i) Control: MacFarlane medium containing normal vitamin solution (5 ng/ml cyanocobalamin); (ii) NB12: no cyanocobalamin added in the vitamin solution used for the MacFarlane medium and (iii) ExtraB12: MacFarlane medium supplemented with high dose of 2500 ng/ml cyanocobalamin final concentration. No B12 was detected in the MacFarlane medium without B12 and in Control medium by UHPLC-DAD method (measured in triplicate; detection limit 30 ng/ml). Therefore, a competitive enzyme immunoassay (RIDASCREEN FAST Vitamin B12, R-Biopharm, Switzerland) was used to quantify B12 content in MacFarlane medium, with and without vitamin mix, quantified as 8.9 ± 0.1 ng/ml and 4.1 ± 0.4 ng/ml, respectively. The detected B12 in the non-supplemented MacFarlane medium might originate from yeast or meat extract added to the medium. The applied concentrations in our *in vitro* colon batch (control 5 ng/ml and ExtraB12 2500 ng/ml) are in the range of what we can expect under average diet conditions [average B12 intake of 4.5 μg/day, (16)] or with extreme B12 supplementation [1500 μg per tablet, (10)]. We therefore considered a small intestine absorption of 50% in the case of average dietary intake (17), and 1% in the case of high dose (11, 17). Consequently, 2.25 μg of B12 under average diet conditions and 5940 μg of B12 under extreme supplementation conditions will arrive in the proximal colon per day. Taking a proximal colon volume of 200 ml (18), and a retention time of 8 h (19), resulting in 600 ml proximal colon suspension per day, which corresponds to the average diet condition of 3.75 ng/ml and with extreme B12 supplementation of 2475 ng/ml.

The flasks were inoculated with the fecal suspension (1% v/v), closed with sterile rubber stoppers and aluminium crimp caps N 20 (Macherey-Nagel AG, Oensingen, Switzerland) and incubated anaerobically at 37^°^C on a shaker (100 rpm) directly in the anaerobic chamber. After 24 h incubation, 1 ml samples were transferred to a sterile Eppendorf tube and centrifuged at 13,000 × *g* for 10 min at 4^°^C. The supernatant was separated from the pellet and filtered through a 0.45 μm nylon membrane filter into a sterile Eppendorf tube. Pellets and supernatants were stored at –20^°^C until further analysis. For each tested condition and fecal microbiota, fermentations were conducted in triplicates.

### Organic acids analysis by high-performance liquid chromatography with refractive index detector

SCFA (acetate, propionate, butyrate, and valerate), intermediate metabolites (lactate, succinate, and formate), and branched-chain fatty acids (BCFA; isobutyrate and isovalerate) were analyzed using HPLC-RI in fecal and fermentation samples. Briefly, 200 mg of feces were weighted and mixed with 600 μl of HPLC eluent (100 mM H_2_SO_4_) in a tube containing one glass bead. After homogenization by vortex, samples were centrifuged for 20 min at 4^°^C (6000 × *g*) and the supernatant was filtered through a 0.2 μm filter into a glass vial and closed with a crimp cap. For fermentation samples, 100 μl of filtered supernatant was diluted with 100 μl filter-sterilized pure water in sterile Eppendorf tubes. The tubes were vortexed, and the content was transferred into a Crimp Neck Vial (BGB Analytik AG, Boeckten, Switzerland). Samples were analyzed using a HPLC (Hitachi LaChrom, Merck, Dietikon, Switzerland) equipped with a precolumn SecurityGuard Cartridges Carbo-H (4 × 3.0 mm; Phenomenex Helvetia GmbH, Basel, Switzerland) connected to a Rezex ROA-Organic Acid H + column (300 × 7.8 mm; Phenomenex Helvetia GmbH, Switzerland) and a refractive index detector (20). Samples (40 μl injection volume) were eluted at 40^°^C under isocratic conditions with 10 mM H_2_SO_4_ at a flow rate of 0.4 ml/min. All metabolites were quantified using external standards (Sigma-Aldrich for all, except valeric acid and formic acid from VWR International AG).

### Vitamin B12 quantification by ultra high-performance liquid chromatography-diode array detector

The protocol for B12 extraction from fecal suspension (1 ml) prepared from homogenized fecal slurry and from fermentation samples (1 ml) was adapted from Chamlagain et al. (21). The protocol includes the addition of potassium cyanide (KCN) to the samples to convert all B12 forms into the stable cyanocobalamin. In fecal suspension samples, total B12 (intracellular and extracellular) was quantified, while in fermentation samples, both intracellular and extracellular B12 was measured separately. For fecal and extracellular quantification, 1 ml extraction buffer (8.3 mM NaOH and 20.7 mM acetic acid) and 10 μl of 1% KCN solution were added to 1 ml sample in 15 ml falcon tubes (Bioswisstec AG, Schaffhausen, Switzerland) and vortexed for 10 s. For intracellular quantification, pellets were resuspended in 1 ml extraction buffer, transferred into 15 ml falcon tubes, 10 μl 1% KCN solution was added, and the tubes were vortexed for 10 s. Samples were then placed in a water bath at 99^°^C for 30 min and afterward ice-cooled for 10 min. Fecal and intracellular samples were centrifuged for 20 min at 4^°^C (10000 × *g*). All samples were filtered through a 0.20 μm nylon membrane filter into brown vials (BGB Analytik AG). All the analyses were carried out under red or dim light conditions to avoid B12 degradation during sample processing.

Cyanocobalamin quantification was performed on a Thermo Scientific Vanquish UHPLC equipped with a Diode Array Detector (DAD) using an Acquity HSS T3 column (2.1 × 1000 mm, 1.8 μm; Waters AG, Baden-Dättwil, Switzerland), connected to ACQUITY UPLC HSS T3 VanGuard Pre-column (100Å, 1.8 μm, 2.1 × 5 mm; Waters AG). The detection was performed at 362 nm. The autosampler was maintained at 12^°^C and the column was operated at 30^°^C. Injection volume was 10 μl. The separation was performed using the mobile phase acetonitrile (A) and MilliQ water (B) both containing 0.1% formic acid using a gradient flow (0.5 ml/min): 0–0.5 min [A 5% and B 95%]; 0.5–5 min [A 40% and B 60%]; 5–6 min [A 40% and B 60%]; 6–10 min [A 95% and B 5%]; 10–11 min [A 5% and B 95%]; 11–15 min [A 5% and B 95%]. The calibration curve was done by measuring cyanocobalamin standards (Sigma-Aldrich) in the 60–4000 ng/ml range. Based on linearity, the limit of detection was 30 ng/ml.

### DNA isolation

DNA was isolated from fermentation pellets obtained from 150 mg homogenized fecal samples and from 1 ml of fermentation sample using the FastDNA Spin Kit for Soil (MP Biomedicals, Illkirch-Graffenstaden, France), following the manufacturer’s protocol. Briefly, samples were placed into 2 ml tubes containing Lysing Matrix E and homogenized (40 s, 6.0 m/s) in a FastPrep^®^ instrument in the presence of MT buffer (lysis solution) and sodium phosphate buffer. Following lysis, samples were centrifuged, and DNA was purified from the supernatant with a silica-based filter in a final volume of 100 μl. DNA was quantified using a Nanodrop^®^ ND-1000 Spectrophotometer and stored at –20^°^C until further analysis.

### Quantification of total bacterial fecal concentration *via* quantitative PCR

Quantitative PCR (qPCR) was used to evaluate the abundance of total bacteria in the fecal samples by quantifying the number of copies of the 16S rRNA gene using primers 338F (5′-ACTCCTACGGGAGGCAGCAG-3′) and 518R (5′-ATTACCGCGGCTGCTGG-3′) (22). Each reaction consisted of 5 μl of 2X SensiFAST SYBR No-ROX Kit master mix (Meridian Bioscience, Cincinnati, OH, USA), 0.5 μl of each forward and reverse primer (final concentration of 0.5 μM each), 1 μl of DNA and 3 μl of nuclease free water. Reactions per sample were performed in triplicates in 96-well plates using a LightCycler 480 qPCR II system (Roche Diagnostics, Rotkreuz, Switzerland) and a two-step program consisting of 3 min of initial denaturation at 95^°^C, followed by 40 cycles of 95^°^C for 5 s and 65^°^C for 30 s. For quantification, a 10-fold dilution series of standards containing a linearized plasmid with the *Escherichia coli* 16S rRNA gene was included in each run. PCR efficiency (%) was calculated from the slope of the standard curve of each qPCR assay. Assays with an efficiency of 80–110% (slope of 3.2–3.9) were included in the data analysis. The qPCR gene copy number was converted to cell concentration by correcting for the median of five 16S rRNA gene copy number per bacterium based on the Ribosomal RNA Database (23).

### 16S rRNA metabarcoding

The microbial composition of fecal inoculum and batch fermentation samples were analysed using 16S rRNA gene sequencing. Briefly, a two-steps PCR approach was used to target the V4 region using primers 515F (5′-GTGCCAGCMGCCGCGGTAA-3′) and 806R (5′-GGACTACHVGGGTWTCTAAT-3′) (Microsynth AG, Balgach, Switzerland) (24). Samples were barcoded using Nextera XT v2 indexes (Illumina, San Diego, CA, USA) and pooled in equimolar concentration. Sequencing was performed with the Illumina MiSeq platform (Genetic Diversity Centre, ETH Zurich, Switzerland) using v2 chemistry supplemented with PhiX 20 pM (10%) and 250 × 2 read length. Raw data were processed using the DADA2 R package (version 1.14.1) (25), to obtain exact Amplicon Sequence Variants (ASVs) using the metabaRpipe R package (26). Briefly, forward and reverse reads were truncated after 260 and 250 nucleotides, respectively. After truncation, reads with expected error rates higher than three and four for forward and reverse reads, respectively, were removed. After filtering, error rate learning, ASV inference and denoising, reads were merged with a minimum overlap of 15 nucleotides. Chimeric sequences were identified and removed. Taxonomy was assigned to ASVs using DADA2 against SILVA database (v138.1) (27, 28). For each fecal donor, enterotype was assigned based on the ASV table at genus level at,^1^ and clustered with Jensen-Shannon divergence (jsd) distance matrix. For fermentation samples, phylogenetic relatedness of ASVs, alpha- (Observed and Shannon indexes) and beta-(Unweighted and Weighted Unifrac distance matrices) diversity analyses were performed using the phyloseq (29) and the DivComAnalyses packages (30) in R Studio (31). ASVs were filtered using the filterByExpr function from the edgeR package (32), and differential analysis of ASVs (NB12 vs. Control, ExtraB12 vs. Control, and ExtraB12 vs. NB12) was performed using the limma-voom pipeline (33) from the limma package (34). ASVs that had an absolute log_2_ fold-change greater than 1 and *P* < 0.001, were considered differentially expressed and dotplots were generated using the ggplot2 package (35).

### Statistical analyses

A non-parametric, Wilcoxon statistical analysis was performed to detect significant differences in alpha diversity between treatments for all donors together, and for each donor separated. The same approach was used for the beta diversity distance matrixes, by PERMANOVA analysis. Both analyses were performed *via* Phyloseq. The statistical analysis for total bacteria, metabolites, and B12 content was done using GraphPad Prism v.9.2.0. An unpaired *t*-test (*P* < 0.05, two-tailed) was performed per each donor to compare different treatments, and the Normality of Residuals was validated using the Shapiro–Wilk test. A two-way ANOVA was performed on the absolute concentration for total SCFA, intermediate, BCFA, and for individual metabolites to determine the main effects of donors and treatments, followed by Tukey’s test (*P* < 0.05) for *post-hoc* analysis to correct for multiple comparisons.

## Results

### Fecal microbiota and B12 content

**FIGURE 1:**
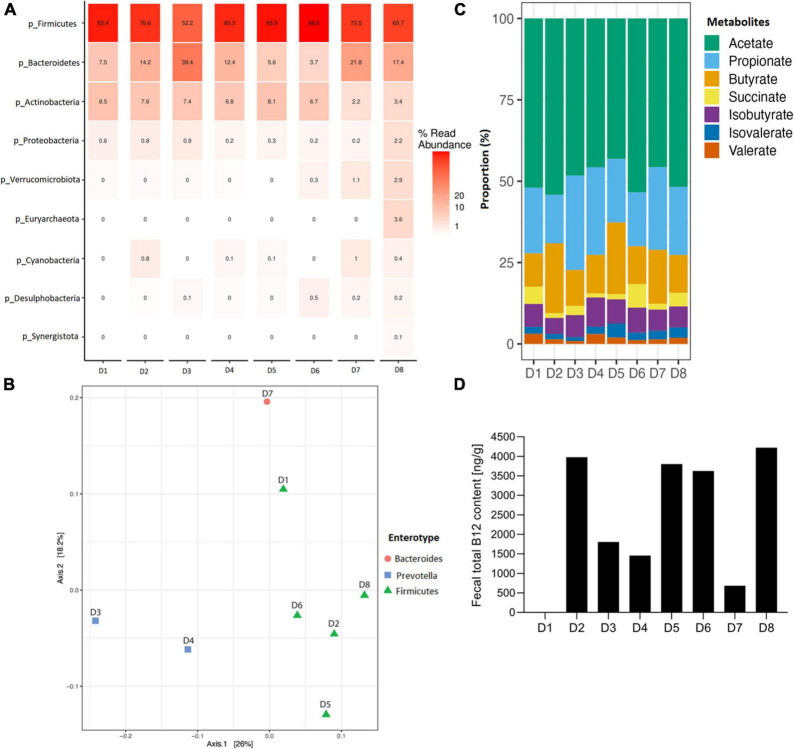
Fecal microbial composition, enterotype clustering, metabolite profile and total B12 content of eight fecal donors (D1 to D8) used in this study before fermentation. **(A)** Relative abundance of fecal microbial communities of each donor at phylum level. **(B)** Donors colored by the enterotype they belong to, with red enterotype 1 (*Bacteroides*), blue enterotype 2 (*Prevotella*) and green enterotype 3 (*Firmicutes*), and clustered with Jensen-Shannon divergence distance matrix. **(C)** Metabolite proportion of each donor. For all donors, lactate and formate were below detection limit, therefore, not shown in the plot. **(D)** Fecal total B12 content (ng/g feces), B12 content for D1 was below detection limit (i.e., 30 ng/ml).

Microbial composition and B12 content in fecal samples of eight adult healthy donors were first measured. *Firmicutes* phylum in all donors ranged from 52.2 to 88.6% relative abundance, as measured by relative abundance on 16S data, with D3 containing also a high *Bacteroidetes* level (39.5% relative abundance; Figure 1A). *Lachnospiraceae* was the most abundant family in all donors (ranging from 33.2 to 46.3%), except for D3 (26.5%), dominated by *Prevotellaceae* at 37.8% (Supplementary Figure 1A). Based on 16S data at the genus level, five donors were assigned to *Firmicutes* (D1, D2, D5, D6 and D8), two to *Prevotella* (D3 and D4), and one to *Bacteroides* (D7) enterotype based on (36) (see text footnote 1) (Figure 1B and Supplementary Figure 1B).

The analysis of fecal SCFAs indicated that D2 and D5 exhibited a butyrogenic profile, having an acetate:propionate:butyrate ratio of 60:16:24 and 51:23:26, respectively (Figure 1C and Supplementary Figure 2). All other donors were propionigenic, with higher propionate than butyrate concentrations in the feces (Figure 1C and Supplementary Figure 2). Fecal total B12 content ranged from 684 ng/g in D7 up to 4222 ng/g in D8, with a median at 2716 ng/g, and was below the detection limit in D1 with a detection limit of the UHPLC-DAD method of 30 ng/ml (Figure 1D). No lactate and formate were detected in fecal samples (limit of quantification 1 mM).

### B12 content in fermentation samples

Batch fermentations of the eight fecal microbial communities grown in buffered MacFarlane medium without added B12 (NB12), and with B12 added in low (Control) or high (ExtraB12) levels were performed for 24 h at 37^°^C under strict anaerobiosis. Extracellular and intracellular B12 were quantified at time 0 (T0) and 24 (T24).

**FIGURE 2:**
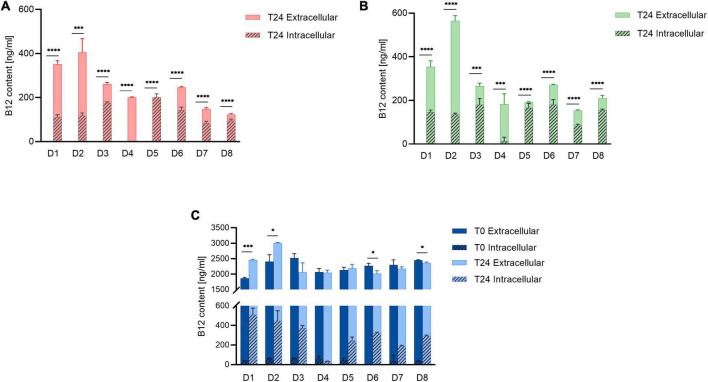
Intracellular and extracellular B12 content of **(A)** Control, **(B)** NB12, and **(C)** ExtraB12 treatments at T0 and T24 for fecal microbiota of eight donors (D1–D8), measured by UHPLC-DAD. B12 content at T0 for control and NB12 treatment was below detection limit (i.e., < 30 ng/ml). Data are mean and standard deviation of three replicates. An unpaired *t*-test was performed on total B12 (sum of intra- and extracellular) to test the significance between T0 and T24, per donor. Asterisk (*) represents significant differences between condition, per each donor (* for *P* < 0.05, or ^***^ for *P* < 0.001, or ^****^ for *P* < 0.0001).

No B12 was detected in Control and NB12 treatment samples for all donors at T0 (Figures 2A,B), while a range from 1860 ± 34 to 2523 ± 141 ng/ml B12 was quantified at T0 in ExtraB12 (Figure 2C). A significant total B12 production was measured for both Control (122 ± 4 to 404 ± 57 ng/ml) and NB12 (152 ± 3 to 564 ± 27 ng/ml) at T24 for all tested donors. Microbiota D3, D5, D6, D7, and D8 had higher levels of intracellular B12 at T24 compared to extracellular (intracellular B12 ranging from 83 ± 9 to 201 ± 15 ng/ml to in Control, from 83 ± 7 to 180 ± 28 ng/ml in NB12, from 188 ± 7 to 370 ± 26 ng/ml in ExtraB12), while D1, D2, and D4 had intracellular B12 content comparatively lower than extracellular (intracellular B12 ranging from 0 to 117 ± 12 ng/ml in Control, from 11 ± 19 to 145 ± 10 ng/ml in NB12, from 31 ± 3 to 506 ± 69 ng/ml in ExtraB12). ExtraB12 treatments showed different responses depending on the donor microbiota after 24 h incubation. A significantly higher concentration of total B12 was observed for D1 and D2, compared to T0, indicating that B12 was produced despite B12 surplus availability. In contrast, a significantly lower content of B12 was quantified at T24 compared to T0 for D6 and D8. For D3, D4, D5, and D7, no significant changes in total B12 content were measured at T24, as compared to T0 (Figure 2C). In all tested conditions, B12 content in D4 was mainly extracellular. For all other donors, a significant higher intracellular B12 content at 24 h was detected in ExtraB12, as compared to NB12 (Supplementary Table 1).

Overall, our data indicate that all gut microbial communities tested could produce B12 after 24 h of fermentation when grown in absence or low amount of B12 in the medium.

### Impact of B12 on gut microbial community growth and composition

Cell numbers were determined by qPCR and microbiota composition was measured at T24 by 16S rRNA sequencing. Total bacteria concentration at T24 were not significantly different among tested conditions and ranged between 10.3 ± 0.15 to 10.8 ± 0.11 log total bacteria in Control samples, 10.5 ± 0.05 to 10.9 ± 0.10 log total bacteria in NB12, and 10.5 ± 0.5 to 11.0 ± 0.2 log total bacteria in ExtraB12 (Supplementary Figure 3).

**FIGURE 3:**
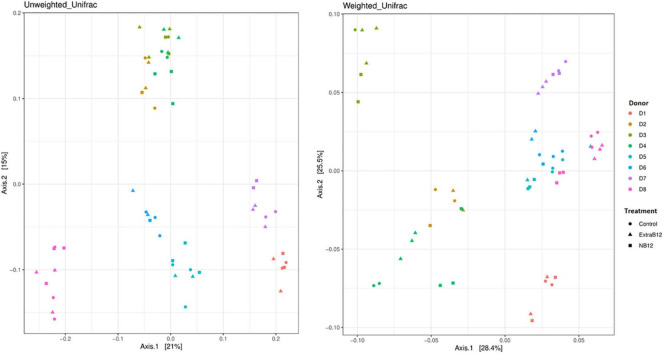
Principal-coordinate analysis plots (PCoA) of unweighted and weighted Unifrac distance matrices of batch fermentation samples of eight microbiota (D1 to D8) after 24 h incubation in different treatments media (Control, NB12, and ExtraB12). The symbols in the PCoA analysis represent the treatments while the colors represent the donors.

**FIGURE 4:**
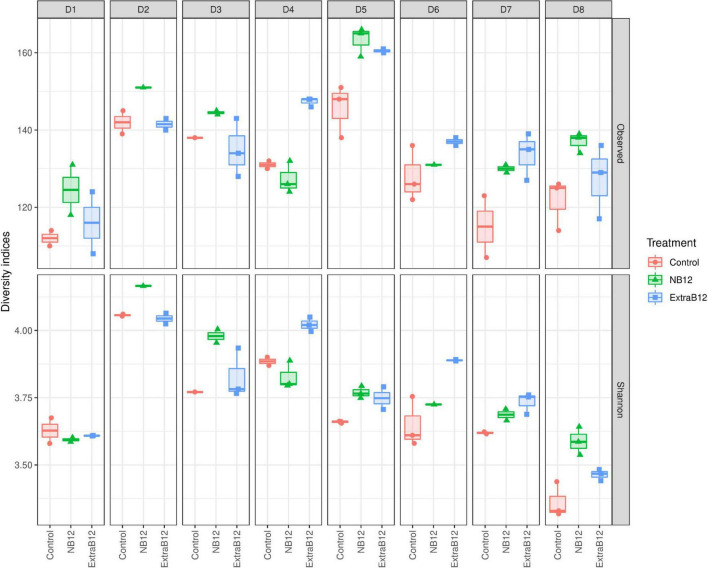
Alpha diversity of fecal microbial communities of eight donors (D1–D8) after 24 h batch fermentation in Control, NB12, and ExtraB12 conditions. Boxplot of alpha diversity of the bacterial communities in the fermentation samples by means of Observed ASVs **(upper panel)** and Shannon **(lower panel)** indexes. The interquartile ranges and boxes, medians (lines in the boxes), and lowest and highest values for the first and third quartiles are presented. Each color represents one treatment as indicated in the legend.

Furthermore, no significant changes in overall microbiota composition in response to treatments were observed (Supplementary Figure 4), as confirmed by beta diversity distance matrices (Figure 3 and Supplementary Figure 5). Neither NB12 nor ExtraB12 significantly impacted alpha diversity among treatments when grouping all donors together (Supplementary Figure 6) and when considering individual donors, compared to Control (Figure 4). Altogether, B12 availability in the medium did not or only show a small impact on total bacterial growth and microbiota composition and diversity, which was rather determined by the initial gut microbial composition of each donor.

**FIGURE 5:**
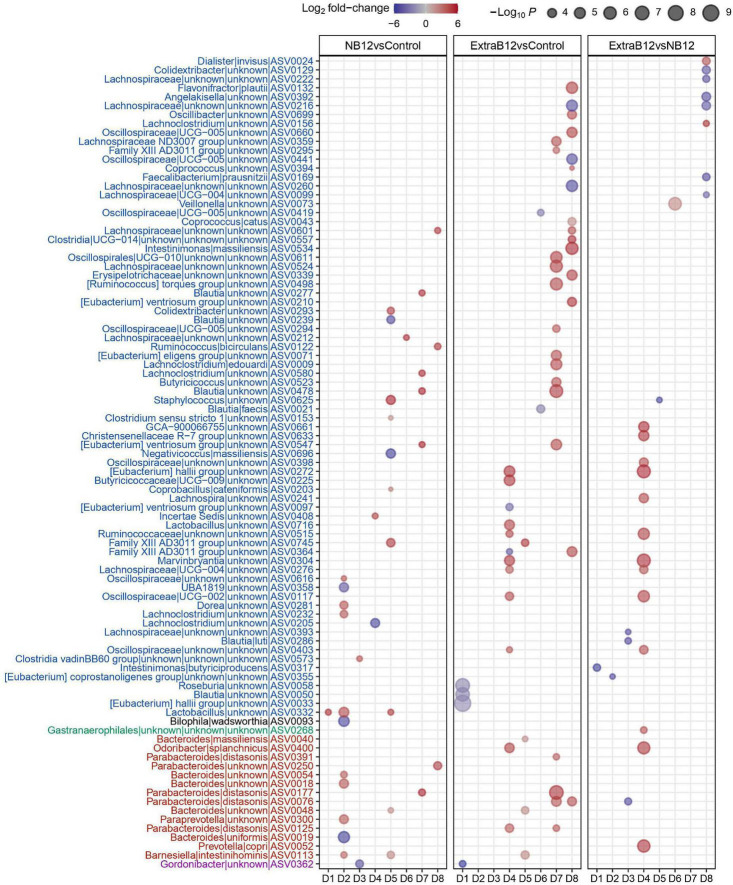
Dot plot of top differentially abundant ASVs between the different batch fermentation conditions per fecal donor microbiota selected based on log_2_ fold change and *P*-value (< 0.001). The color of the dot represents the change (blue indicates a decrease in the treatment; red indicates an increase in the treatment), and the size of the dot represents the significance, the bigger the size, the higher the significance is. The x-axis represents the donors (D1–D8), and the y-axis represents the different genus and ASVs grouped by phyla, into different colors (Blue = *Firmicutes*, Black = *Desulphobacteria*, green = *Cyanobacteria*, red = *Bacteroidetes*, and pink = *Actinobacteria*).

A total of 91 ASVs for all donors changed in abundance at different B12 concentrations, of which 81% belonged to the *Firmicutes*, and 15% to the *Bacteroidetes* phylum (Figure 5). Most ASV changes were found in only one microbiota, implying donor-specific changes at the ASV level upon B12 treatments. However, few ASVs responded in more than one microbiota for each of the treatments, as compared to Control. Specifically, for NB12 ASV0332 (*Lactobacillus*) in D1, D2, D5, and ASV0113 (*Barnesiella intestinihominis*) in D2 and D5 responded with increased abundance, as compared to Control. In ExtraB12, two ASVs both assigned to *Parabacteroides distasonis* (ASV0076 and ASV0125) increased in abundance in D7, D8 and D4, D7, respectively, as compared to Control. When NB12 was compared to ExtraB12, none of the ASV were shown to respond in more than one donor (Figure 5).

Overall, observed changes at the individual ASV level did not result in changes in microbiota community structure, as supported by alpha and beta diversity analyses.

### Impact of B12 on metabolite production

**FIGURE 6:**
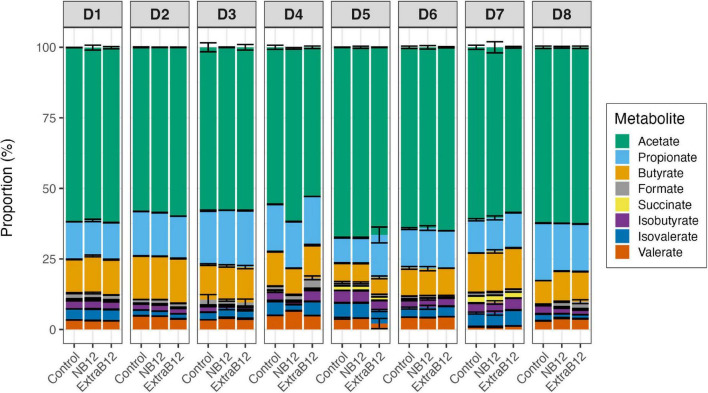
Proportion of metabolites produced after 24 h batch fermentations of eight fecal donor microbiota (D1 to D8) in three treatments conditions (Control, NB12, and ExtraB12). Proportion of acetate, propionate, butyrate, formate, lactate, succinate, isobutyrate, isovalerate, and valerate with respect to the total metabolites produced after fermentation. Average and standard deviation of three replicates. Lactate was below the limit of quantification (1 mM) for all donors microbiota.

B12 is required as a cofactor by gut microbes for various biological processes, including acetate production *via* the Wood-Ljungdahl pathway, and for the conversion of succinate to propionate. After 24 h, the proportion of total metabolites did not change in NB12 or ExtraB12, as compared to Control, for all tested microbiota (Figure 6), independently of the initial donor fecal metabolite profile (Figure 1C).

When considering all donors together, significant variations in individual metabolite production due to the donor’s effect were reported by two-way ANOVA (Supplementary Table 2), while none of the treatments impacted total metabolite production. Overall, a significant increase in propionate in ExtraB12 was reported by ANOVA, as compared to NB12 (Supplementary Table 3). Furthermore, a higher concentration of succinate was observed in Control, compared to ExtraB12 (Supplementary Table 3).

**FIGURE 7:**
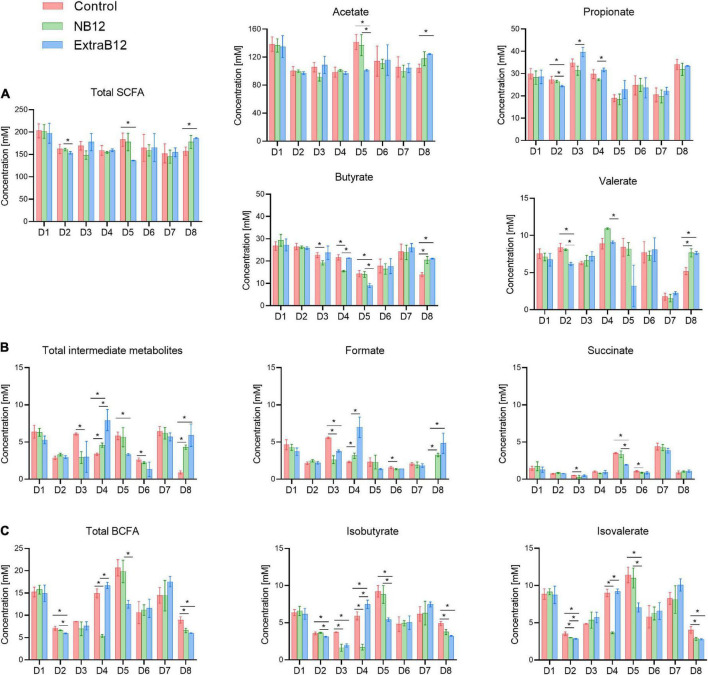
Metabolite production after 24 h batch fermentation of eight fecal donor microbiota (D1 to D8) under different treatment condition (Control, NB12 and ExtraB12). **(A)** SCFA (acetate, propionate, butyrate and valerate); **(B)** BCFA (isobutyrate and isovalerate) and **(C)** intermediate metabolites (formate and succinate) are shown. Data are represented as average and standard deviation of three replicates. Per each measured metabolite and donor, an asterisk (*) and the bar indicate significant differences (*P* < 0.05) between treatments. Lactate was below the limit of quantification (1 mM) for all microbiota.

Donor-dependent metabolic responses were observed in individual donors (Figure 7). However, there were some metabolic changes observed in more than one donor. Briefly, D3 and D4 responded to NB12 with a significant decrease in butyrate (–3.4 ± 0.8 mM and –6.2 ± 0.7 mM, respectively) and isobutyrate (–2.1 ± 0.2 mM, –4.2 ± 0.3 mM), compared to Control. The same donors reported a significant increase in propionate (+ 8.35 ± 1.8 mM, + 4.5 ± 0.5 mM, respectively) when NB12 was compared to ExtraB12 (Figure 7A). On total intermediate metabolites, D3 and D6 showed a significant decrease (–3.16 ± 0.58 and –0.42 ± 0.14 mM, respectively) in response to NB12, compared to Control, while the opposite trend was observed in ExtraB12 for D4 (4.58 ± 0.83 mM) and D8 (5.01 ± 0.88 mM), due to formate increase (Figure 7B). In response to NB12, total BCFA decreased in D4 (–9.54 ± 0.61 mM) and D8 (–2.35 ± 0.49 mM), as compared to control, while in response to ExtraB12, BCFA decreased in D5 (–8.16 ± 1.43 mM) and D8 (–2.93 ± 0.40 mM) (Figure 7C).

To conclude, overall, the treatments with different doses of B12 had no effect on total metabolite production, though minor effects were observed on propionate and succinate production (Supplementary Tables 2, 3 and Figure 7). No lactate was detected in fermentation samples (limit of quantification 1 mM).

## Discussion

A significant amount of unabsorbed dietary vitamin B12 is expected to reach the colon (9, 17), thus being available for the densely populated gut bacterial community. Although it is known that specific gut species are B12 auxotrophs (37–41), whether the overall gut microbial community uses exogenous B12 for growth and metabolism or relies on endogenously produced B12 for potential cross-feeding between prototrophs and auxotrophs remains unclear. *In vitro* fermentation models were used in this study to accurately estimate B12 consumed or produced by healthy adult fecal microbiota independent of host absorption. In particular, such models allow to study different treatments in parallel, eliminating host factors and providing a good estimation of complete balances on the produced metabolites (13).

The enterotype clustering of fecal samples confirmed that the donors belonged to *Firmicutes*, *Prevotella*, and *Bacteroides* enterotype groups (36). The representativity of the donor for the target population was also confirmed by the metabolic profiling, which comprised both butyrogenic and propionigenic donors. The ratio of acetate, propionate, and butyrate quantified in the feces here was similar to a previously reported ration for adult gut microbiota (42).

The detected fecal B12 concentration in the samples is within the range of what was previously reported. Fecal B12 content is influenced by donor and dietary intake, including supplements. A previous study reported an average of 1309 ng total B12/g wet weight of adult human feces, upon a daily intake of < 25 μg B12 a day, whereas participants who took supplements with 1 to 2 mg a day, displayed very high B12 levels between 4820 and 5417 ng total B12/g wet weight of feces (9).

When gut microbial communities were cultivated for 24 h in control and in low or no-B12 medium, a production of B12 by all tested gut microbiota was observed, demonstrating that human gut microbial communities have an intrinsic capability to produce B12, an essential vitamin for both the host and certain microbes. Interestingly, some donors further accumulated significantly higher B12 intracellularly when a high level was added to the medium, compared to Control and NB12. Furthermore, the presence of extracellular B12 suggests that microbially-produced B12 was released during fermentation *in vitro*, likely due to cell-lysis, and might be available for potential cross-feeding to the auxotrophic species of the community. Different cobamides uptake systems have been identified in Gram-positive and Gram-negative bacteria, such as *Bacteroides* spp. that have up to four cobamides uptake systems (43, 44). However, it is not yet known how intracellularly synthesized cobamides are released into the gut environment to enable exchange between producers and consumers (44). Our data suggest that fecal communities can function without exogenous B12, and that the presence of prototrophic bacteria could supply enough B12 for the community. Therefore, as indicated before, the predicted 20% B12 prototrophs of the human gut microbiota could possibly provide B12 for the remaining 80% of auxotrophic taxa (43).

Gut microbial composition analysis further supported this assumption, as all donors were mostly dominated by *Firmicutes* (> 50% relative abundance), followed by *Bacteroidetes* (up to 39.5% in D3). A previous study reported that half of the *Firmicutes* and *Bacteroidetes* members are B12 prototrophs, and harbour the full set of genes required for *de novo* B12 biosynthesis (12). Similarly here, at the genus level, a high relative abundance of predicted prototrophs was observed in all donors, including *Blautia* (> 7%), *Faecalibacterium* (> 5%), *Anaerostipes* (> 1.5%), and *Bacteroides* (> 1.2%), among others (12). In line, these taxa were also detected after 24 h *in vitro* fermentation in all conditions, indicating their growth during batch fermentation.

To further confirm that gut communities do not need exogenous B12, we measured total bacteria as well as microbiota community composition in no, low, and high-B12 addition after 24 h of incubation. Indeed, tested healthy adult gut communities did not rely on exogenous B12 as total bacteria and community composition did not differ in no and high-B12 supplementation, compared to control. In line with this, a previous study showed that gut microbial communities from healthy adults remained stable *in vitro* in response to extremely high doses of exogenous B12 (2500 or 75000 ng/ml adenosylcobalamin) (45). Interestingly, the same was observed *in vivo* using healthy mice, either fed with no or an extremely high dose (200 mg/kg) of cyanocobalamin (46), or with a single oral dose of 3.94 μg/ml (equivalent to a 5 mg dose for humans) (47). In comparison, the Recommended Dietary Intake of B12 for human adults (2.4 μg/day) is three orders of magnitude lower. However, in another study with cobalamin deficient patients, B12 intake in the methylcobalamin and cyanocobalamin forms, appeared to shift gut microbiome composition and activity *in vitro* after 7 days (48). In line with those observations, while B12 supplementation did not alter the gut microbiota composition of mice under healthy conditions, it contributed to differential microbial responses after the induction of experimental colitis in mice (46).

At the metabolic level, ExtraB12 treatment showed a significant increase in propionate production when compared to NB12, considering all donors together. Such an increase was driven by D3 and D4, which were, among the six propionigenic donor microbiota, the only *Prevotella* enterotypes. B12 is necessary for the synthesis of propionate to catalyze the formation of propionyl-CoA, an important step in the conversion of succinate to propionate (49, 50).

Overall, the highest variability observed between microbiota was driven by the donor rather than the applied treatments. This implies that B12 supplementation has a minor impact on complex community structure and metabolic activity with respect to the donor’s effect.

This study is preliminary with only eight healthy adult donors. Therefore, the number and enterotype distribution of donors should be increased for drawing a definitive biological conclusion. Moreover, B12 as cyanocobalamin was tested here, and it is known that cobamides with different lower ligands are not equivalent for different taxa (43, 51). The presence of cobamide forms may have different modulation effects on the gut microbiota compared to cyanocobalamin because various bacteria can remodel alternative cobamides into cobalamin (5). Furthermore, we used a simple batch fermentation model suitable for a short period of time and to test the growth dynamic and metabolic potential of fecal microbiota.

## Conclusion

Our study shows that human gut microbial communities produce B12 and sustain growth and metabolic activity and potential B12 cross-feeding to auxotrophic taxa of the community. Furthermore, our results suggest supplementation of different levels of B12 in the medium did not largely impact gut microbiota growth, composition, and metabolic activity during the 24 h fecal batch fermentation of different feces composition. The above findings should be confirmed by increasing the number of donors. Moreover, further studies based on a more physiologically relevant, long-term continuous fermentation model of the human gut should be performed to investigate the effect of supplementation and depletion of B12. Lastly, whether microbially produced B12 in the gut is used by the host is not yet known.

## Data availability statement

The data presented in the study are deposited in the ENA repository, accession number PRJEB57690, https://www.ebi.ac.uk/ena/browser/view/PRJEB57690.

## Author contributions

AGr, AGe, and CL conceived the research. PM and PK performed the experiments. PK developed the UHPLC-DAD method. PK and BP quantified vitamin B12. AGr, AGe, PK, BP, and CL analysed and interpreted the data. All authors contributed to the manuscript writing and reviewing.

## Footnotes

1
http://enterotypes.org/
