# Supplementary material for: Healthy adult gut microbiota sustains its own vitamin B12 requirement in an in vitro batch fermentation model
Source: Front Nutr. 2022 Dec 1;9:1070155. doi: 10.3389/fnut.2022.1070155 (PMC9751363; doi:10.3389/fnut.2022.1070155)
Supplement: Supplementary file 1 [file Data_Sheet_1.docx]

**Supplementary Figures and Tables**

**
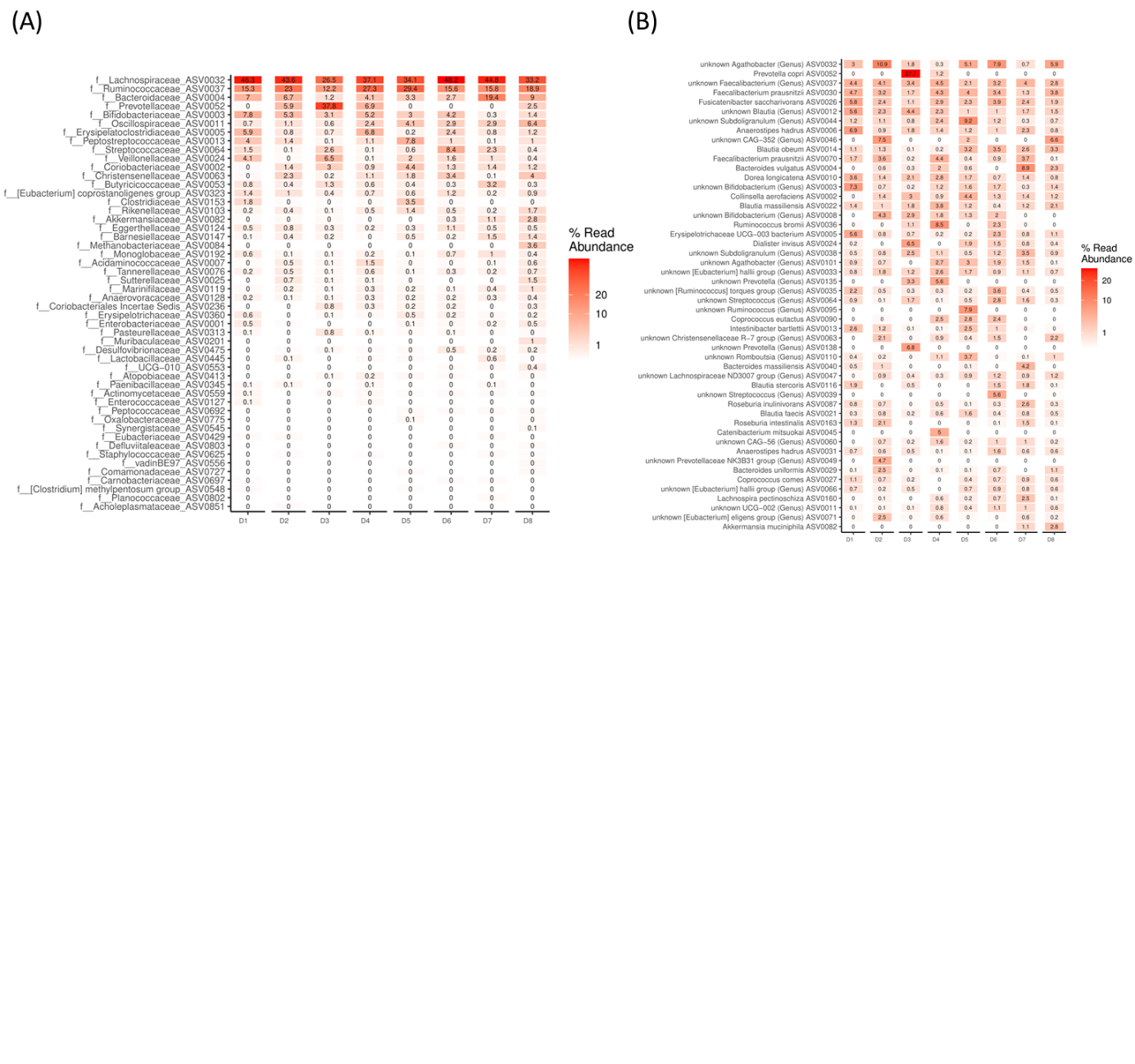
**

**Figure S1. Microbial composition of eight fecal donors (D1 to D8) used in this study, measured by 16S rRNA metabarcoding.** Relative abundance of fecal microbial communities for each donor before fermentation at (**A**) family and (**B**) genus level**,** represented as mean of triplicate of the top 50 most abundant.


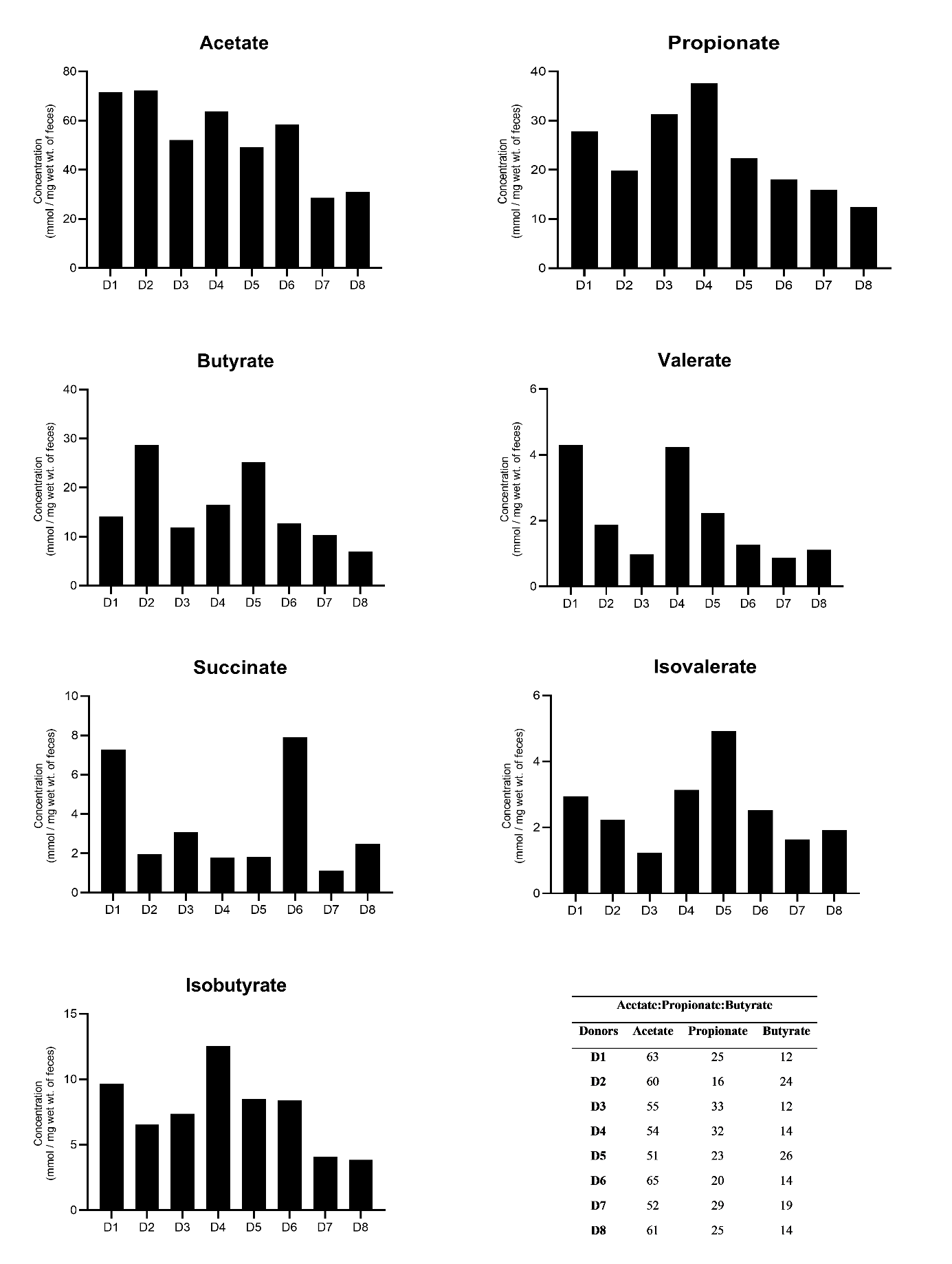


**Figure S2. Metabolite profile of eight fecal donors (D1 to D8) used in this study, measured by HPLC-RI.** Acetate, propionate, butyrate, valerate, succinate, isovalerate and isobutyrate measured as mmol per mg of wet weight of feces. Per each SCFA, the ratio (as %) is shown in the table (acetate:propionate:butyrate). Lactate and formate were not detected for all donors; therefore, they are not shown.


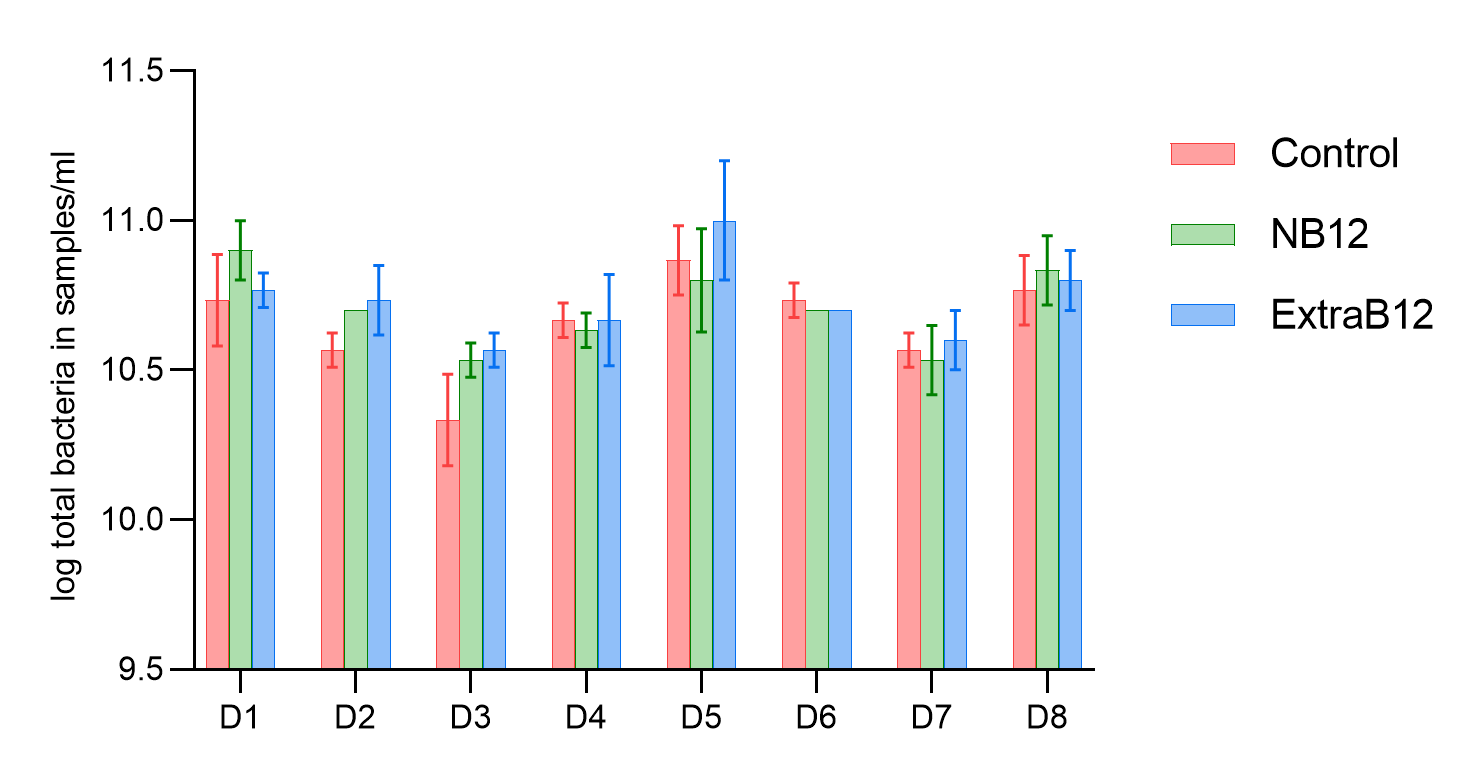


**Figure S3. Total bacteria quantified by qPCR in samples of fermentation samples of eight donors (D1-D8) in all tested conditions (Control, NB12, and ExtraB12) after 24 h of batch fermentations**. Values are mean and standard deviation of triplicates quantification. No standard deviation was detected for D2 (NB12) and D6 (NB12 and ExtraB12) since there was no variation between triplicate data values.

**Figure S4**. **Microbial composition of 24 h fermentation in different B12 medium for eight fecal donors (D1 to D8), measured by 16S rRNA metabarcoding.** Relative abundance of fecal microbial communities for each donor at genus level after 24 h batch incubation in Control, NB12 and ExtraB12 fermentation condition, represented as mean of triplicate of the top 50 most abundant.


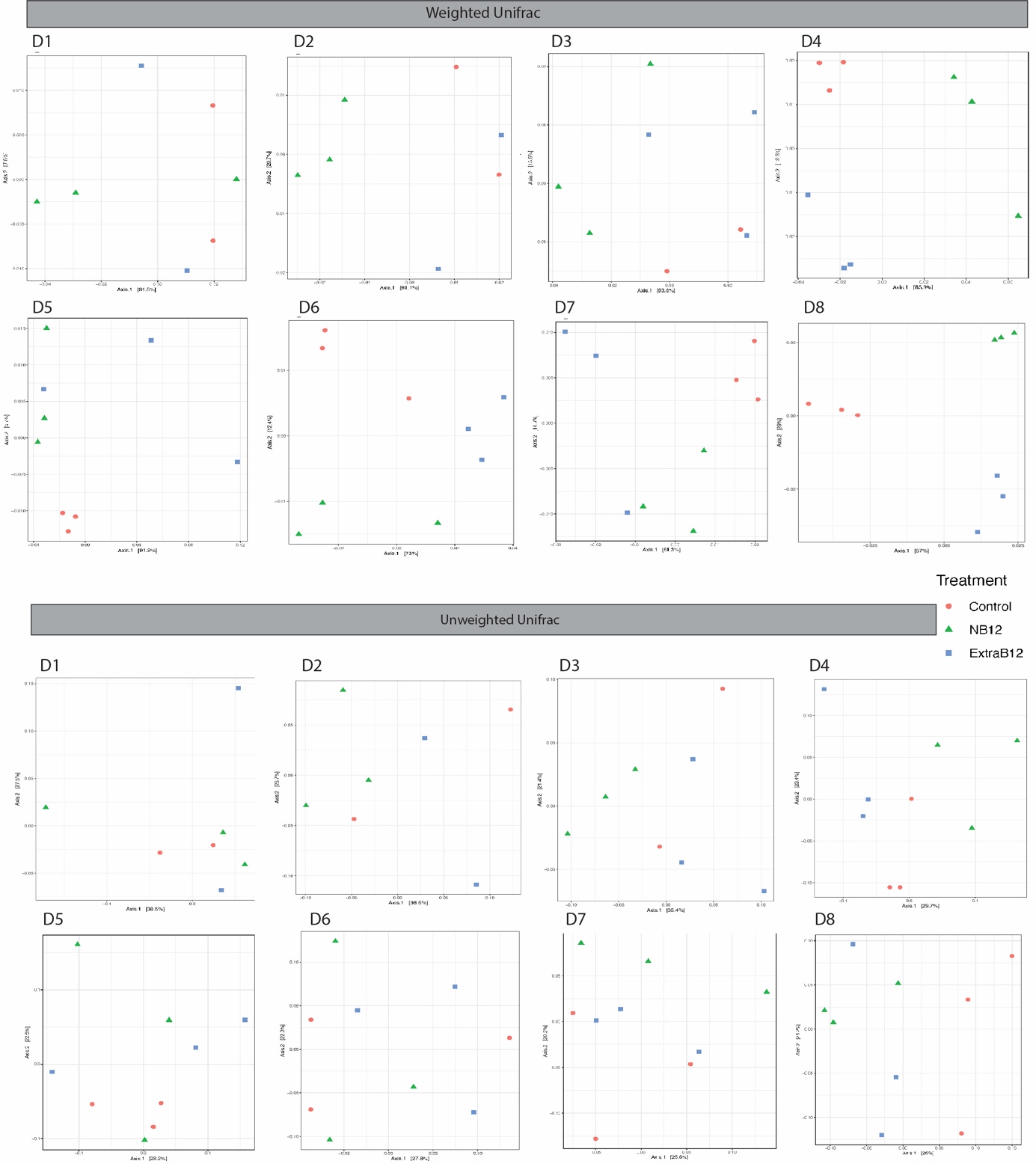


**Figure S5**. **Beta diversity matrix of each fecal donor microbiota (D1 to D8) based on weighted and unweighted Unifrac distance matrix after 24 h of batch incubation under different fermentation condition (Control, NB12 and ExtraB12).**

**Figure S6. Alpha diversity of each treatment for all donors together, as measured by Observed and Shannon index after 24 h of batch fermentations in three conditions (Control, NB12 and ExtraB12).**

| **Donor** | **Comparison** | **Mean Difference ± SEM** | **P value Summary** | **P Value** |
| --- | --- | --- | --- | --- |
| D1 | **NB12 vs. Control** | **33.53 ± 9.157** | ***** | **0.0215** |
|  | **ExtraB12 vs. control** | **394.5 ± 40.77** | ******* | **0.0006** |
|  | **ExtraB12 vs. NB12** | **361.0 ± 40.72** | ******* | **0.0009** |
| D2 | NB12 vs. Control | 17.93 ± 8.350 | ns | 0.0982 |
|  | **ExtraB12 vs. control** | **326.6 ± 61.80** | ****** | **0.0061** |
|  | **ExtraB12 vs. NB12** | **308.7 ± 61.47** | ****** | **0.0074** |
| D3 | NB12 vs. Control | 5.067 ± 16.78 | ns | 0.7778 |
|  | **ExtraB12 vs. control** | **195.0 ± 15.66** | ******* | **0.0002** |
|  | **ExtraB12 vs. NB12** | **189.9 ± 22.60** | ****** | **0.0011** |
| D4 | NB12 vs. Control | 11.50 ± 11.50 | ns | 0.3739 |
|  | **ExtraB12 vs. control** | **31.97 ± 1.732** | ******** | **<0.0001** |
|  | ExtraB12 vs. NB12 | 20.47 ± 11.63 | ns | 0.1532 |
| D5 | NB12 vs. Control | -35.53 ± 14.43 | ns | 0.0695 |
|  | ExtraB12 vs. control | 42.20 ± 23.73 | ns | 0.1499 |
|  | **ExtraB12 vs. NB12** | **77.73 ± 24.82** | ***** | **0.0351** |
| D6 | NB12 vs. Control | 38.17 ± 16.45 | ns | 0.0811 |
|  | **ExtraB12 vs. control** | **177.2 ± 11.19** | ******** | **<0.0001** |
|  | **ExtraB12 vs. NB12** | **139.1 ± 15.86** | ******* | **0.0009** |
| D7 | NB12 vs. Control | 0.9 ± 6.721 | ns | 0.8999 |
|  | **ExtraB12 vs. control** | **105.9 ± 6.932** | ******* | **0.0001** |
|  | **ExtraB12 vs. NB12** | **105.0 ± 6.257** | ******** | **<0.0001** |
| D8 | **NB12 vs. Control** | **59.87 ± 5.842** | ******* | **0.0005** |
|  | **ExtraB12 vs. control** | **196.6 ± 7.377** | ******** | **<0.0001** |
|  | **ExtraB12 vs. NB12** | **136.7 ± 6.742** | ******** | **<0.0001** |

**Table S1. A summary table showing comparison of intracellular B12 after 24 h fermentations between each treatment (Control, NB12, and ExtraB12) for all donors (D1-D8).** An unpaired *t*-tes was performed on intracellular B12 for to test the significance changes between each condition. Significant changes are marked in bold. An Asterisk (*) represents significant differences (* for *P* < 0.05, or **** for *P* < 0.0001), while “ns” stands for non-significant. All analysis were performed with three sample size for each condition.

**Table S2. Two-way ANOVA analysis performed to determine main effect of donors and treatments on each fermentation metabolite.** Column F (DFn, DFd) represents F-statistics, where DFn stands for degree of freedom numerator and DFd stands for degree of freedom denominator. Significant changes are marked in bold. An Asterisk (*) represents significant differences (* for *P* < 0.05, or **** for *P* < 0.0001), while “ns” stands for non-significant. Total SCFA represents sum of acetate, propionate, butyrate and valerate, BCFA represents sum of isobutyrate and isovalerate and total intermediate metabolites represents sum of formate and succinate.

| **Metabolite** | **Source of Variation** | **% Total variation** | **F (DFn, DFd)** | **P value** | **P value summary** |
| --- | --- | --- | --- | --- | --- |
| Total SCFA | Donors  Treatment | 45.90  0.4 | **F (7, 61) = 7.447**  F (2, 61) = 0.2279 | **<0.0001**  0.7969 | ********  ns |
| Total BCFA | Donors  Treatment | 71.86  2.226 | **F (7, 61) = 24.17**  F (2, 61) = 2.620 | **<0.0001**  0.0801 | ********  ns |
| Total intermediate metabolites | Donors  Treatment | 5031  0.4129 | **F (7, 60) = 8.727**  F (2, 60) = 0.2507 | **<0.0001**  0.7791 | ********  ns |
| Acetate | Donors | 59.35 | **F (7, 61) = 12.85** | **<0.0001** | ******** |
|  | Treatment | 0.2515 | F (2, 61) = 0.1906 | 0.8270 | ns |
|  |  |  |  |  |  |
| Propionate | Donors | 79.32 | **F (7, 60) = 37.97** | **<0.0001** | ******** |
|  | Treatment | 2.338 | **F (2, 60) = 3.678** | **0.0274** | ***** |
|  |  |  |  |  |  |
| Butyrate | Donors | 76.32 | **F (7, 61) = 29.69** | **<0.0001** | ******** |
|  | Treatment | 0.6863 | F (2, 61) = 0.9344 | 0.3984 | ns |
|  |  |  |  |  |  |
| Formate | Donors | 31.24 | **F (7, 59) = 4.220** | **0.0008** | ******* |
|  | Treatment | 4.552 | F (2, 59) = 2.153 | 0.1252 | ns |
|  |  |  |  |  |  |
| Succinate | Donors | 92.95 | **F (7, 61) = 139.0** | **<0.0001** | ******** |
|  | Treatment | 0.7744 | **F (2, 61) = 4.054** | **0.0222** | ***** |
|  |  |  |  |  |  |
| Isobutyrate | Donors | 65.77 | **F (7, 61) = 18.54** | **<0.0001** | ******** |
|  | Treatment | 3.185 | F (2, 61) = 3.142 | 0.0503 | ns |
|  |  |  |  |  |  |
| Isovalerate | Donors | 76.47 | **F (7, 61) = 30.11** | **<0.0001** | ******** |
|  | Treatment | 1.483 | F (2, 61) = 2.043 | 0.1385 | ns |
|  |  |  |  |  |  |
| Valerate | Donors | 74.96 | **F (7, 61) = 27.91** | **<0.0001** | ******** |
|  | Treatment | 1.596 | F (2, 61) = 2.079 | 0.1338 | ns |

**Table S3. Tukey’s multiple comparison test performed to determine main effect of treatments on each fermentation metabolite after 24 h fermentations.** Significant changes are marked in bold. An Asterisk (*) represents significant differences (* for *P* < 0.05), while “ns” stands for non-significant. Total SCFA represents sum of acetate, propionate, butyrate and valerate, BCFA represents sum of isobutyrate and isovalerate and total intermediate metabolites represents sum of formate and succinate.

| **Metabolite** | **Tukey's multiple comparisons test** | **Mean Difference** | **P value Summary** | **Adjusted P Value** |
| --- | --- | --- | --- | --- |
| Total SCFA | Control vs. NB12  Control vs. ExtraB12  NB12 vs. ExtraB12 | 3.195  1.079  -2.116 | ns  ns  ns | 0.7851  0.9733  0.9014 |
| Total BCFA | Control vs. NB12  Control vs. ExtraB12  NB12 vs. ExtraB12 | 1.722  0.7130  -1.009 | ns  ns  ns | 0.0665  0.6221  0.3902 |
| Total intermediate metabolites | Control vs. NB12  Control vs. ExtraB12  NB12 vs. ExtraB12 | -0.2173  -0.3003  -0.0830 | ns  ns  ns | 0.8708  0.7732  0.9800 |
| Acetate | Control vs. NB12 | 1.761 | ns | 0.8646 |
|  | Control vs. ExtraB12 | 1.909 | ns | 0.8464 |
|  | NB12 vs. ExtraB12 | 0.1480 | ns | 0.9990 |
| Propionate | Control vs. NB12 | 1.474 | ns | 0.1260 |
|  | Control vs. ExtraB12 | -0.56 | ns | 0.8019 |
|  | **NB12 vs. ExtraB12** | **-2.034** | ***** | **0.0271** |
| Butyrate | Control vs. NB12 | 0.3620 | ns | 0.8866 |
|  | Control vs. ExtraB12 | -0.6936 | ns | 0.6513 |
|  | NB12 vs. ExtraB12 | -1.056 | ns | 0.3746 |
| Formate | Control vs. NB12 | -0.3223 | ns | 0.7042 |
|  | Control vs. ExtraB12 | -0.8657 | ns | 0.1063 |
|  | NB12 vs. ExtraB12 | -0.5435 | ns | 0.4033 |
| Succinate | Control vs. NB12 | 0.0604 | ns | 0.8007 |
|  | **Control vs. ExtraB12** | **0.2623** | ***** | **0.0224** |
|  | NB12 vs. ExtraB12 | 0.2028 | ns | 0.0985 |
| Isobutyrate | Control vs. NB12 | 0.9177 | ns | 0.0503 |
|  | Control vs. ExtraB12 | 0.4957 | ns | 0.3806 |
|  | NB12 vs. ExtraB12 | -0.4219 | ns | 0.4948 |
| Isovalerate | Control vs. NB12 | 0.8042 | ns | 0.1321 |
|  | Control vs. ExtraB12 | 0.2173 | ns | 0.8608 |
|  | NB12 vs. ExtraB12 | -0.5869 | ns | 0.3420 |
| Valerate | Control vs. NB12 | -0.4017 | ns | 0.5102 |
|  | Control vs. ExtraB12 | 0.3421 | ns | 0.6196 |
|  | NB12 vs. ExtraB12 | 0.7438 | ns | 0.1124 |
